# Antibacterial properties of the pituitary adenylate cyclase-activating polypeptide: A new human antimicrobial peptide

**DOI:** 10.1371/journal.pone.0207366

**Published:** 2018-11-21

**Authors:** Somia Debbabi, Marie-Christine Groleau, Myriam Létourneau, Chitra Narayanan, Laura-Lee Gosselin, Mustapha Iddir, Jacinthe Gagnon, Nicolas Doucet, Eric Déziel, David Chatenet

**Affiliations:** INRS–Institut Armand-Frappier, Université du Québec, Ville de Laval, Canada; Nanyang Technological University, SINGAPORE

## Abstract

The Pituitary Adenylate Cyclase-Activating Polypeptide (PACAP), a polycationic, amphiphilic and helical neuropeptide, is well known for its neuroprotective actions and cell penetrating properties. In the present study, we evaluated the potent antibacterial property of PACAP38 and related analogs against various bacterial strains. Interestingly, PACAP38 and related analogs can inhibit the growth of various bacteria including *Escherichia coli* (JM109), *Bacillus subtilis* (PY79), and the pathogenic *Burkholderia cenocepacia* (J2315). Investigation of the mechanism of action suggested that a PACAP metabolite, identified as PACAP(9–38), might indeed be responsible for the observed PACAP38 antibacterial action. Surprisingly, PACAP(9–38), which does not induce haemolysis, exhibits an increased specificity toward *Burkholderia cenocepacia* J2315 compared to other tested bacteria. Finally, the predisposition of PACAP(9–38) to adopt a π-helix conformation rather than an α-helical conformation like PACAP38 could explain this gain in specificity. Overall, this study has revealed a new function for PACAP38 and related derivatives that can be added to its pleiotropic biological activities. This innovative study could therefore pave the way toward the development of new therapeutic agents against multiresistant bacteria, and more specifically the *Burkholderia cenocepacia* complex.

## Introduction

Increased bacterial resistance to available antibiotics is a situation of global concern[1]. Based on their broad spectrum of activities and usually non-immunogenic action, antimicrobial peptides (AMPs) isolated from various species including plants, mammals, insects, and marine invertebrates[2], represent promising alternatives to traditional antibiotics. Natural antimicrobial peptides are relatively short polypeptides (fewer than 60 amino acid residues) that generally possess a positive net charge and amphipathic properties upon interaction with membranes[3]. Based on their structural (linear or helical) and biochemical properties (presence or absence of cysteine or over-representation of proline, arginine, tryptophan and histidine), antimicrobial peptides can be divided into at least five different groups[4]. Several companies have intensified their efforts to commercially develop AMPs, many of which are now in clinical trials[5]. However, their clinical use for human treatment remains limited to topical applications since AMPs are usually toxic or metabolically unstable when injected into the bloodstream[6]. Recently, the discovery of bacteria resistant to colistin, an antibiotic of last resort for many multidrug resistant bacteria was reported[7], further prompting the discovery of new treatments to fight a growing number of infections evading the latest generations of antibiotics.

The pituitary adenylate cyclase-activating polypeptide is a 38 amino acid peptide (PACAP38) that belongs to the vasoactive intestinal polypeptide (VIP)-glucagon-growth hormone releasing factor-secretin superfamily[8]. This peptide, well known for its pleiotropic activities in human, mediates its biological activities through the activation of three G protein-coupled receptors, named PAC1, VPAC1, and VPAC2[8]. Notably, PACAP38 and its analogs, mostly synthetic agonists, are currently regarded as promising candidates for the treatment of neurodegenerative disorders and brain trauma[9]. The recent discovery of intracellular PACAP38 receptors in the brain and the testis as well as the physicochemical characteristics of PACAP38, *i*.*e*. an extended amphipathic α-helix containing basic residues, prompted us to evaluate the propensity of PACAP38 to cross the plasma membrane in a receptor-independent manner[10]. We demonstrated the cell penetrating properties of PACAP38, which led to the development of a potent PACAP38-based cell penetrating peptide, [Arg^17^]PACAP(11–38), with no cytotoxic effect on various eukaryotic cells including CHO-K1[11]. Based on this unique property, and since most AMPs exhibit similar physicochemical and structural characteristics, we hypothesize that PACAP38 and its related derivatives can exert antimicrobial activities and therefore represent a new template for the development of a new class of innovative AMPs.

In this study, the antibacterial potential of PACAP38 as well as several selected derivatives was tested against various Gram-positive and Gram-negative bacteria. Our results demonstrate the potent antibacterial activity of PACAP38 against various bacterial strains, helping us identify a lead template, *i*.*e*. PACAP(9–38), that appears to be specific against *Burkholderia cenocepacia*, an epidemic pathogen of cystic fibrosis patients.

## Materials and methods

### Materials

Fmoc-protected amino acids, Rink-amide AM resin and BOP (benzotriazol-1-yloxy)tris(dimethylamino)phosphonium hexafluorophosphate) reagents were purchased from Chem-Impex (Wood Dale, IL, USA). Solvents for solid phase peptide synthesis and purification were obtained from Tekniscience (Terrebonne, QC, CAN) whereas trifluoroacetic acid (TFA) was from PSIG (Montreal, QC, CAN). Na^125^I was purchased from Perkin Elmer (Montreal, QC, CAN). Other chemicals as well as cell culture media were obtained from Sigma-Aldrich (Mississauga, ON, CAN) and Fisher Scientific (Nepean, ON, CAN). The culture media for bacteria were purchased from BD Difco (Mississauga, ON, CAN).

### Peptide synthesis

The synthesis of PACAP38 and related analogs have been reported elsewhere[8]. Magainin-2 was synthesized by solid phase peptide chemistry using the Rink-amide AM resin as a solid support and standard Fmoc/tBu chemistry. Couplings of the protected amino acids were mediated by BOP (3 eq) and DIPEA (4.5 eq) in DMF for 1h. Coupling efficiency was monitored with the qualitative ninhydrin test and a 3-equivalent excess of the protected amino acids based on the original substitution of the resin (0.53 mmol.g^-1^) was used in most cases. Fmoc removal was achieved with 20% piperidine in DMF for 20 min. Peptide cleavage was achieved at room temperature using a mixture of TFA/ethanedithiol/phenol/water (92/2.5/3/2.5) for 3h. The diethyl ether-precipitated crude peptides were purified on a preparative RP-HPLC using a Phenomenex C_18_ Gemini column and the collected fractions were analyzed concomitantly by analytical RP-HPLC, performed on a Phenomenex C_18_ Jupiter column, and MALDI-TOF mass spectrometry using α-cyano-4-hydroxycinnamic acid as matrix (Voyager DE, Applied Biosystems). Fractions corresponding to the desired product and a purity higher than 95% were finally pooled and lyophilized.

### Minimal inhibitory concentration (MIC) measurements

Assays were performed according to the CLSI microdilution method [12]. Briefly, the bacterial strains were inoculated and allowed to reach an OD_600_ of 0.08–0.13 prior to dilution to 10^6^ CFU/mL in Mueller-Hinton broth (MHB). A bacterial suspension (50 μL) was then added to each well of a 96-well microplate containing serially diluted peptide solutions (50 μL in MHB) and incubated at 37˚C for 20h. A well containing sterilized medium was used as a sterility control and another well containing no added peptide served as a growth control. The MIC endpoint, expressed in μg/mL, represents the lowest concentration of PACAP38 or related analogs at which no bacterial growth was observed after the incubation time, as determined by OD measurements.

### Membrane and cytoplasmic proteins extraction

*Burkholderia cenocepacia* strains J2315 and K56-2 membrane and cytoplasmic protein extraction was performed as previously described with some modifications[13]. Bacteria were grown in 8 mL of Tryptic Soy Broth (TSB) until they reached an OD_600_ of 2 and then PACAP38 (200 μg/mL) was added to the cultures for 1h. The treated culture was then centrifuged at 7,000 x *g* for 15 min at 4˚C and the supernatant discarded. The resulting pellet was resuspended in 500 μL of cold Tris-HCl (10 mM; pH 8) containing 20% sucrose (w/v), frozen at -20˚C and then thawed to facilitate bacterial cells disruption. Following the addition of DNase (50 μg/mL), bacteria were lysed by FastPrep-24 (MPBio) using 0.1 mm glass beads (4.5 m/s, 60s). The suspension was then centrifuged at 10,000 x *g* for 30 min at 4˚C and the supernatant, containing the cytoplasmic proteins, recovered and kept at 4˚C until use. The beads were then mechanically removed and the remaining pellet was ultracentifuged in a sucrose gradient (50% and 70%) at 100,000 x *g* overnight at 4˚C in order to separate the inner (eluting between 20% and 40% of the sucrose gradient) and outer (eluting between 50% and 70% of the sucrose gradient) bacterial membranes. The resulting membrane fractions were collected in a minimum volume of water and kept at 4˚C until use. A similar procedure was used to extract membrane and cytoplasmic proteins from *Bacillus subtilis* strains PY79 and 2597[14]. Briefly, *Bacillus subtilis* strains were grown in 8 mL of Tryptic Soy Broth (TSB) to an OD_600_ of 2 and then PACAP38 (200 μg/ml) was added to the cultures for 1h before centrifugating at 8,000 x *g* for 10 min at 4˚C; finally, the pellet was washed twice with water. Bacterial cell lysis was then performed by FastPrep and the different protein extracts were isolated as described above, and kept at 4˚C until use.

Finally, the presence of PACAP or its metabolites in those different protein extracts was investigated by Matrix-Assisted Laser Desorption/Ionization Time-of-Flight (MALDI-TOF) mass spectrometry using α-cyano-4-hydroxycinnamic acid as the matrix.

### Haemolytic activity

All experimental procedures were performed in accordance with regulations and ethical guidelines and approved by the institutional committee of the Institut National de la Recherche Scientifique-Institut Armand-Frappier. Blood from healthy human volunteers was collected into sodium citrate-buffered vacutainers. Blood donations were obtained from informed and consenting individuals according to institutionally approved procedures. Red blood cells were pelleted (700 x *g*; 10 min), washed three times with 9 volumes of PBS by centrifugation (700 x *g*; 10 min), and then resuspended in PBS. Cell suspensions (1 mL of final suspension) were incubated with different concentrations of peptide for 30 min or 3h, ranging from 10^−9^ to 10^−5^ M (4.5 ng/mL to 45 μg/mL for PACAP38 and 3.7 ng/mL to 37 μg/mL for PACAP (9–38)), at 37˚C with occasional mixing by inversion. PBS-incubated red blood cells were considered as negative control and maximum lysis of erythrocytes was obtained by incubating the cells with 0.1% (v/v) Triton X-100. At the end of the incubation period, cells were pelleted (700 x *g*, 10 min) and 100 μL of each supernatant was used to evaluate the release of hemoglobin by measuring the absorbance at 545 nm. Results were obtained from 3 independent experiments performed in triplicate.

### Cell culture

Chinese hamster ovary (CHO) cells stably and individually expressing human receptor PAC1, VPAC1 or VPAC2 were grown in Ham’s F12 medium supplemented with 10% of fetal bovine serum (FBS), 2 mM L-glutamine, 100 UI/mL of penicillin and streptomycin (P/S) and 400 μg/mL of G418. The cell line was maintained at 37˚C in a humidified atmosphere of 5% CO_2_ and passages were performed by trypsinization when cells reached about 80% confluence.

### Radioligand binding assay

Acetylated PACAP27 was radioiodinated using the chloramine-T oxidation technique and purified on a *Sep*-*Pak* C_18_ cartridge (Waters, Milford, MA, USA) [15]. Binding assays were performed using CHO cells stably transfected with the human PAC1, VPAC1 or VPAC2 receptor isoform. Cells were seeded at a density of 150,000 cells/well in 24-well plates. The next day, cells were washed with binding buffer (0.1% BSA, 25 mM Tris–HCl, 25 mM MgCl_2_, pH 7.4) for 10 min at room temperature. The solution was replaced with a fresh solution containing 0.05 nM of ^125^I-Ac-PACAP27 and increasing concentrations of peptide (10^−14^ to 10^-5^M). After 2h of incubation at room temperature, cells were washed twice with the binding buffer and then lysed with sodium hydroxide (0.1 M). Cell-bound radioactivity was quantified using a γ-counter. Results were expressed as percentage of specific binding of ^125^I-Ac-PACAP27. Non-specific binding was determined in the presence of PACAP38 (10^-5^M).

### Circular dichroism analysis

Circular dichroism (CD) spectra were recorded at room temperature from 200 to 250 nm, using a 1 mm optical path length with a 0.1 nm step, a 1 nm bandwidth, and an integration time of 4s on a Jasco J-815 Circular Dichroism (CD) Spectropolarimeter (Easton, MD, USA). Each spectrum represents the mean of three scans corrected for solvent contribution. A digital low-pass filter was used as a smoothing routine. Peptides were dissolved in 20 mM KH_2_PO_4_ (pH 7.0) at a final concentration 200 μg/mL.

### Molecular dynamic (MD) simulations of membrane-bound PACAP38 and PACAP(9–38)

MD simulations of the DPPC membrane-bound conformations of PACAP38 and PACAP(9–38) peptides were performed to characterize the secondary structure properties of the membrane-bound peptides. All simulations were performed under constant pressure, temperature and number of molecule (NPT) conditions, using the GROMACS simulation package v4.5.4.[16] Peptides were simulated using the GROMOS96 53A6 forcefield[17], while the lipids were described using the modified Berger force field parameters[18] for use with the GROMOS96 53A6 force field. The peptides were aligned parallel to the surface of the lipid bilayer and solvated with SPC water. Chloride ions were added to neutralize the charge of the system. The simulation system consisted of 2 x 64 DPPC lipids, one peptide (PACAP38 or PACAP(9–38)), and ~ 7000 water molecules. The initial system box size was set to 6.4 nm x 6.4 nm x 9.5 nm. The systems were first energy minimized using the steepest descent method, followed by short equilibration runs before the long MD simulations. Two sets of simulations were performed for each of the two membrane-bound peptide systems (PACAP38 and PACAP(9–38)), corresponding to a total of 4 x 300 ns runs.

The temperature was set to 323 K using the Nose-Hoover thermostat with a coupling constant of 0.5 ps. The pressure was applied semi-isotropically and maintained using the Parinello-Rahman pressure coupling. All bonds and angles were constrained using the LINCS algorithm[19] and the particle mesh Ewald (PME)[20] method with a grid spacing of 0.16 nm was used for long-range interactions. The α-helical coordinates of micelle-bound PACAP38 (PDB: 2D2P), determined by NMR, were used as the starting conformation of the peptide. The structure of PACAP(9–38) was obtained by truncating PACAP38 to remove the first eight residues. The DPPC coordinates were taken from http://moose.bio.ucalgary.ca. Analysis of secondary structure propensity of the peptides along the simulation trajectory were performed using DSSP[21].

## Results and discussion

### Antibacterial activity of PACAP38 and PACAP-based derivatives

The physico-chemical characteristics of PACAP38, *i*.*e*. extended α-helix containing 11 basic residues[8], as well as its cell-penetrating properties(10), a characteristic often associated with antimicrobial activity, prompted us to evaluate the propensity of PACAP to exert antibacterial activity. Hence, PACAP38 (**1**) and its related analogs were initially tested using the CLSI microdilution method for their capacity to inhibit the growth of various bacterial strains. Selected PACAP38 analogs were chosen based on their improved metabolic stability in human plasma and against DPP-IV, *i*.*e*. N-hexanoyl-PACAP38 (**2**), [Ala^15^]PACAP38 (**3**), [Ala^20^]PACAP38 (**4**), [Ala^21^]PACAP38 (**5**), [Ala^14,20^]PACAP38 (**6**)[22], or their inability to bind and activate PACAP38 cognate receptors, *i*.*e*. [Tic^6^]PACAP38 (**7**) and [Tic^6^]PACAP27 (**8**)[23]. As shown in Table 1, compounds **1–8** have a very weak or no antibacterial activity against *Pseudomonas fluorescens* (MF37), *Pseudomonas aeruginosa* (PA14), *Burkholderia thailandensis* (ATCC700388), *Serratia marcescens* (ATCC14756), *Bacillus cereus* (ATCC11778), and two *Staphylococcus aureus* strains (ATCC6538 and Newman), all showing a minimal inhibitory concentration (MIC) over 200 μg/mL. In the same assay, the reference AMP Magainin 2 (**9**), which assumes an amphiphilic helix when bound to acidic phospholipids just like PACAP38[24], was also unable to inhibit the growth of those strains (Table 1). Interestingly, we uncovered seven bacteria, including *Pseudomonas putida* (KT2440), *Escherichia coli* (JM109), *Burkholderia cenocepacia* (J2315), *Bacillus circulans* (LSPQ3543), *Actinobacillus pleuropneumoniae* (4074), *Bacillus subtilis* (PY79), *Bacillus velezensis*, and *Bacillus amyloliquefaciens* (LMG22478), which are sensitive to PACAP38 and/or its related analogs (Table 1). Notably, *B*. *cenocepacia* (J2315) and *B*. *subtilis* (PY79) are especially sensitive to PACAP38 and its derivatives, displaying MIC values ranging from 2 to 40 μg/mL.

**Table 1:** Minimal inhibitory concentration (MIC)^a^ of PACAP and related analogs on various bacterial strains.

	**1**	**2**	**3**	**4**	**5**	**6**	**7**	**8**	**9**
***Pseudomonas putida* (KT2440)**	113	77	93	56	186	113	33	>160	>200
***Pseudomonas* fluorescens (MF37)**	>200	>200	>200	112	>200	>200	>200	>160	>200
***Pseudomonas aeruginosa* (PA14)**	>200	>200	>200	112	>200	>200	>200	>160	>200
***Escherichia coli* (JM109)**	>200	116	93	28	112	37	>200	>160	165
***Burkholderia cenocepacia* (J2315)**	10	10	9	5	20	8	2	>160	>200
***Burkholderia thailendensis* (ATCC700388)**	>200	>200	>200	>200	>200	>200	>200	>160	>200
***Serratia marcescens* (ATCC14756)**	>200	>200	>200	>200	>200	>200	>200	>160	>200
***Bacillus circulans* (LSPQ3543)**	94	58	21	47	84	94	6	>160	>200
***Bacillus cereus* (ATCC11778)**	>200	>200	>200	>200	>200	>200	>200	>160	>200
***Actinobacillus pleuropneumoniae***	7.5	232	112	>200	>200	>200	>200	>160	>200
***Bacillus subtilis* (PY79)**	28	12	5	3	42	3.5	7	>160	154
***Bacillus velezensis***	76	87	33	61	168	52	71	>160	>200
***Bacillus amyloliquefaciens***	42	26	10	7	20	9	21	>160	134
***Staphylococcus aureus (*ATCC6538#P)**	>200	>200	>200	>200	>200	>200	>200	>160	>200
***Staphylococcus aureus* (Newman)**	>200	>200	>200	>200	>200	>200	>200	>160	>200

^a^ Minimum inhibitory concentration (MIC) represents the lowest concentration of an antimicrobial that will inhibit the visible growth of a microorganism after overnight incubation. Concentrations are expressed in μg/ml. Experiments were done at least in triplicates.

*In vivo*, PACAP exists in two isoforms of 27 and 38 amino acids that share structural, physicochemical and biological properties[8]. Both isoforms, containing between 5 and 11 basic residues, adopt an amphipathic helical conformation, starting around their ninth residue[9]. It is well known that the C-terminal domain of PACAP38 is involved in the stabilization of the α-helix[8]. In the present work, removal of the C-terminal domain of compound **7** produces a PACAP analog, *i*.*e*. [Tic^6^]PACAP27 (**8**), which loses the ability to reduce bacterial growth (MIC > 160 μg/mL). Since both derivatives, *i*.*e*. [Tic^6^]PACAP38 and [Tic^6^]PACAP27, possess an extended C-terminal helix, the number of positively charged residues decorating those analogs (5 residues for compound **8** and 11 for **7**) might be an essential trait to exert their antibacterial activity.

We next evaluated the specificity of PACAP activity against additional strains from the same species. While PACAP and its derivatives are highly active against strain J2315 of *B*. *cenocepacia*, they were inactive against four other strains belonging to the same species (Table 2). Additional strains of *P*. *aeruginosa* and *E*. *coli* were also evaluated with results mostly in accordance with the previously observed effects (Tables 1 and 2).

**Table 2:** MIC^a^ of PACAP and related analogs on homologous bacterial strains.

	**1**	**2**	**3**	**4**	**5**	**6**	**7**	**9**
**Pseudomonas aeruginosa (ATCC33350-Sero-2)**	>200	>200	>200	>200	>200	>200	>200	>200
***Escherichia coli* ATCC25922**	>200	>200	>200	>200	>200	>200	>200	>200
***Escherichia coli* (O157 :H7)**	>200	>200	>200	168	>200	113	>200	>200
***Burkholderia cenocepacia* (K56-2)**	>200	>200	>200	>200	>200	>200	>200	>200
***Burkholderia cenocepacia* (LMG18829)**	>200	>200	>200	>200	>200	>200	>200	>200
***Burkholderia cenocepacia* (LMG19240)**	>200	>200	>200	>200	>200	>200	>200	>200
***Burkholderia cenocepacia* (CEP0511 Gvar3b)**	>200	>200	>200	>200	>200	>200	>200	>200
***Bacillus subtilis* (2597)**	>200	154	111	168	56	187	>200	>200

^a^Minimum inhibitory concentration (MIC) represents the lowest concentration of an antimicrobial that will inhibit the visible growth of a microorganism after overnight incubation. Concentrations are expressed in μg/ml. Experiments were performed at least in triplicate.

### Insight into the PACAP38 mode of action

The known cell penetrating properties of **1** as well as its ability to interact with biological membranes prompted us to evaluate its propensity to interact with the bacterial membrane and to penetrate inside bacteria. To do so, **1** was incubated with various bacterial strains for 1h, allowing the peptide to enter the cells. No bacterial death was observed after this incubation period (data not shown). The bacterial cells were then fractionated into compartments and the presence of **1** or its metabolites in the outer/inner membranes or the cytoplasmic fraction was investigated by mass spectrometry (Table 3).

**Table 3:** Mass spectrometry analysis of bacterial compartments following incubation with PACAP38.

Cell fraction	Observed mass (Da)	Sequence	Calculated mass (Da)
***Bacillus subtilis* (PY79)**			
**Membrane**	4534.1	HSDGIFTDSYSRYRKQMAVKKYLAAVLGKRYKQRVKNK	4534.3
**Cytoplasm**	4536.6	HSDGIFTDSYSRYRKQMAVKKYLAAVLGKRYKQRVKNK	4534.3
	3667.9	SYSRYRKQMAVKKYLAAVLGKRYKQRVKNK	3661.3
***Bacillus subtilis* (2597)**			
**Membrane**	4534.1	HSDGIFTDSYSRYRKQMAVKKYLAAVLGKRYKQRVKNK	4534.3
**Cytoplasm**	-	_-_	-
			
***Burkholderia cenocepacia* (J2315)**			
**Outer membrane**	4533.2	HSDGIFTDSYSRYRKQMAVKKYLAAVLGKRYKQRVKNK	4534.3
	3666.6	SYSRYRKQMAVKKYLAAVLGKRYKQRVKNK	3661.3
**Inner membrane**	-	-	-
**Cytoplasm**	4542.3*	HSDGIFTDSYSRYRKQMAVKKYLAAVLGKRYKQRVKNK	4534.3
	3663.3	SYSRYRKQMAVKKYLAAVLGKRYKQRVKNK	3661.3
***Burkholderia cenocepacia* (K56-2)**			
**Outer membrane**	4536.3	HSDGIFTDSYSRYRKQMAVKKYLAAVLGKRYKQRVKNK	4534.3
	3669.2	SYSRYRKQMAVKKYLAAVLGKRYKQRVKNK	3661.3
**Inner membrane**	-	-	-
**Cytoplasm**	4543.8*	HSDGIFTDSYSRYRKQMAVKKYLAAVLGKRYKQRVKNK	4534.3
**Cell fraction**	Observed mass (Da)	Sequence	Calculated mass (Da)
***Bacillus subtilis* (PY79)**			
**Membrane**	4534.1	HSDGIFTDSYSRYRKQMAVKKYLAAVLGKRYKQRVKNK	4534.3
**Cytoplasm**	4536.6	HSDGIFTDSYSRYRKQMAVKKYLAAVLGKRYKQRVKNK	4534.3
	3667.9	SYSRYRKQMAVKKYLAAVLGKRYKQRVKNK	3661.3
***Bacillus subtilis* (2597)**			
**Membrane**	4534.1	HSDGIFTDSYSRYRKQMAVKKYLAAVLGKRYKQRVKNK	4534.3
**Cytoplasm**	-	_-_	-
			
***Burkholderia cenocepacia* (J2315)**			
**Outer membrane**	4533.2	HSDGIFTDSYSRYRKQMAVKKYLAAVLGKRYKQRVKNK	4534.3
	3666.6	SYSRYRKQMAVKKYLAAVLGKRYKQRVKNK	3661.3
**Inner membrane**	-	-	-
**Cytoplasm**	4542.3*	HSDGIFTDSYSRYRKQMAVKKYLAAVLGKRYKQRVKNK	4534.3
	3663.3	SYSRYRKQMAVKKYLAAVLGKRYKQRVKNK	3661.3
***Burkholderia cenocepacia* (K56-2)**			
**Outer membrane**	4536.3	HSDGIFTDSYSRYRKQMAVKKYLAAVLGKRYKQRVKNK	4534.3
	3669.2	SYSRYRKQMAVKKYLAAVLGKRYKQRVKNK	3661.3
**Inner membrane**	-	-	-
**Cytoplasm**	4543.8*	HSDGIFTDSYSRYRKQMAVKKYLAAVLGKRYKQRVKNK	4534.3

*Methionine oxidation

As described above, PACAP38 can reduce the growth of *B*. *subtilis* strain PY79 but not of strain 2597 (Tables 1 and 2). Mass spectrometry analyses revealed that **1** (PACAP38) is found associated with the bacterial membrane in both strains but only present within the cytoplasmic fractions of PY79. Interestingly, a presumptive metabolite, identified as PACAP(9–38), was only observed associated with the sensitive PY79 strain. Furthermore, a similar pattern was also observed with the *B*. *cenocepacia* strains. While PACAP38 was observed on the outer membrane and within the cytoplasm of both *B*. *cenocepacia* strains, PACAP(9–38) was again only present within the cytoplasmic fractions of the sensitive J2315 strain. Hence, presence of the PACAP(9–38) metabolite in the cytoplasm seems to correlate with sensitivity to the inhibitory activity of PACAP38. The inability of **1** to reduce the growth homologous strains of *Bacillus subtilis* PY79 and *Burkholderia cenocepacia* J2315 might therefore point toward the existence of different proteases in these bacterial strains. Hence, while *B*. *cenocepacia* K56-2 and J2315 belong to the same clonal complex, they have several differences in their genomes and phenotypes[25]. Indeed, although there is only a draft genome of K-56-2 available (www.burkholderia.com), several differences in annotated proteases and peptidases are noted. Further experiments will be needed to confirm our assumption.

### PACAP(9–38): A potentially selective antimicrobial peptide

To confirm the bactericidal activity of this PACAP38-derived metabolite, the analog was prepared by solid phase peptide synthesis. The pharmacological profile of this synthetic PACAP(9–38) was evaluated using three CHO cell lines respectively co-expressing the human PAC1, VPAC1 or VPAC2 receptors. As depicted in earlier publications, the N-terminal domain of PACAP plays an essential role for binding affinity and biological activity[8, 23, 26, 27]. Hence, compared to the native PACAP38, PACAP(9–38) is characterized by a dramatic reduction of its binding affinity towards all PACAP receptors (Table 4), much like PACAP(11–38)(11). PACAP(9–38) was then tested against several PACAP38-sensitive bacterial strains. Unexpectedly, PACAP(9–38) was only able to reduce, with high efficiency (MIC: 19 μg/mL), the growth of strain J2315 of *B*. *cenocepacia* (Table 5), leaving unaffected several PACAP38-sensitive strains including *P*. *putida* (KT2440), *E*. *coli* (JM109), and *B*. *subtilis* (PY79).

**Table 4:** Binding affinity of PACAP38 and PACAP(9–38).

	HPLC^a^	MS^b^ calc	MS^c^ found	PAC1		VPAC1		VPAC2	
				IC_50_ (nM)^d^	pIC_50_	IC_50_ (nM)^d^	pIC_50_	IC_50_ (nM)^d^	pIC_50_
PACAP38	98%	4534.3	4534.9	4.6 (2.7–7.7)	8.34 ± 0.11	3.6 (2.2–5.7)	8.45 ± 0.09	18 (4–78)	7.74 ± 0.28
PACAP(9–38)	98%			> 10^−6^	5.87 ± 0.19	> 10^−6^	5.29 ± 0.37	> 10^−6^	5.77 ± 0.13

^a^Percentage of purity determined by HPLC using the eluent system: A = H_2_O (0.1% TFA) and B = 60% CH3CN/40% A with a gradient slope of 1% B/min, at a flow rate of 1 mL/min on a Vydac C_18_ column. Detection at 229 nm.

^b^Theorical monoisotopic molecular weight as calculated with ChemDraw Ultra 7.0.1.

^c^m/z value assessed by MALDI-TOF-MS.

^d^IC_50_ represents the concentration at 50% binding inhibition. Values in parentheses are 95% confidence limits.

**Table 5:** MIC^a^ values of PACAP(9–38) (10) against PACAP-sensitive bacteria.

	10
***Burkholderia cenocepacia* (J2315)**	19
***Burkholderia cenocepacia* (K56-2)**	>183
***Pseudomonas putida* (KT2440)**	>183
***Escherichia coli* (JM109)**	>183
***Bacillus subtilis* (PY79)**	>183
***Bacillus subtilis* (2597)**	>183
***Bacillus amyloliquefaciens***	>183
***Actinobacillus pleuropneumoniae***	>183

^a^Minimum inhibitory concentration (MIC) represents the lowest concentration of an antimicrobial that will inhibit the visible growth of a microorganism after overnight incubation. Concentrations are expressed in μg/ml. Experiments were performed at least in triplicate.

**Fig 1:**
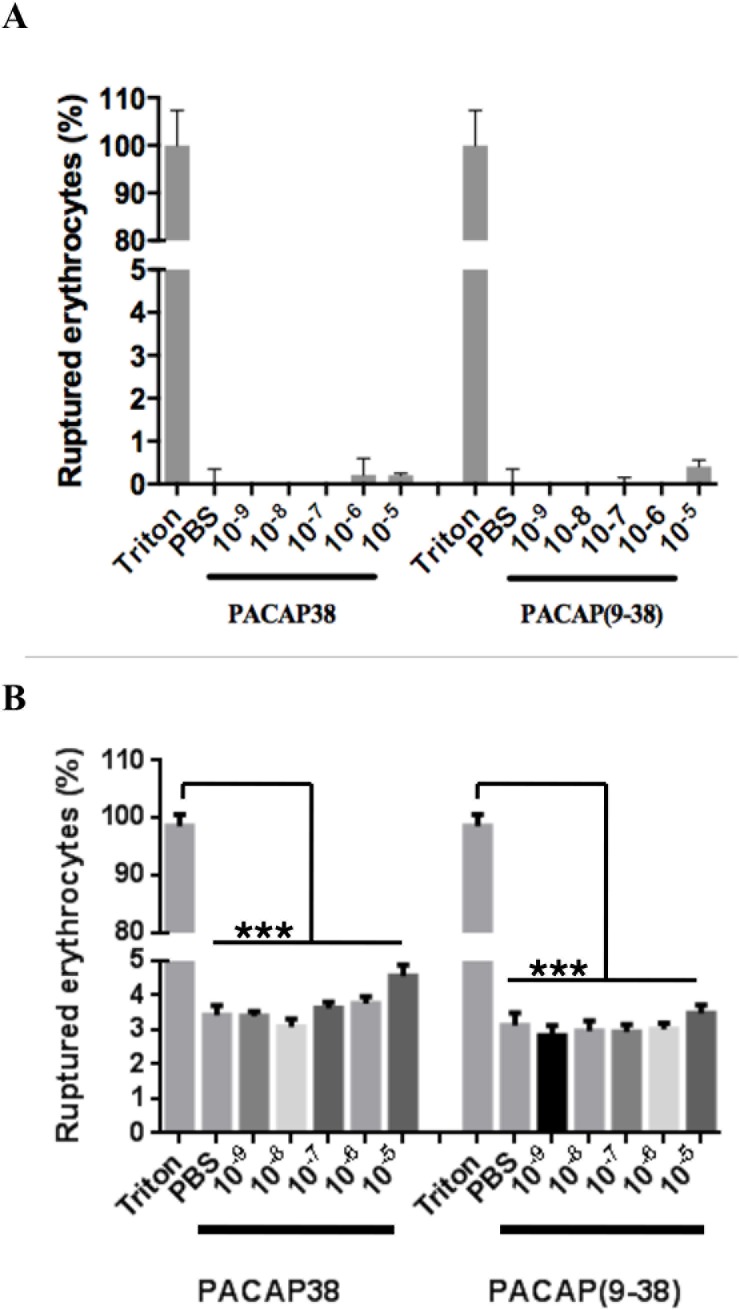
**Haemolytic activity of PACAP38 and PACAP(9–38) performed after 30 min (A) and and 3h (B) incubation with various concentrations of PACAP38 or PACAP(9–38).** Statistical analysis was performed by using ANOVA, followed by a Dunnett’s multiple comparison test, and differences were considered significant when ***P < 0.001.

The percentage of haemolysis caused by various concentrations (4.5 ng/mL—45 μg/mL) of PACAP38 and (3.7 ng/mL—37 μg/mL) of PACAP(9–38) over 30 min or 3h at 37°C is shown in Fig 1 At 37 or 45 μg/mL, PACAP38 and PACAP(9–38) do not exert a very significant haemolytic activity. While less prominent than *P*. *aeruginosa*-associated infection among cystic fibrosis patient, *B*. *cenocepacia*-related infection in such patients provokes a severe decline in lung function that might end up into a life-threatening systemic infection known as cepacia syndrome [28]. Treatment of such infection is extremely difficult as *B*. *cenocepacia* is highly resistant to multiple antibiotics or antibiotic combination [29]. With its apparent selectivity against *B*. *cenocepacia* J2315 and a potentially good therapeutic index, PACAP(9–38) represents a suitable candidate for the development of antimicrobial agents against cystic fibrosis-associated infection involving *B*. *cenocepacia*.

### Relationships between the secondary structure and biological activity of PACAP38 and PACAP(9–38)

**Fig 2:**
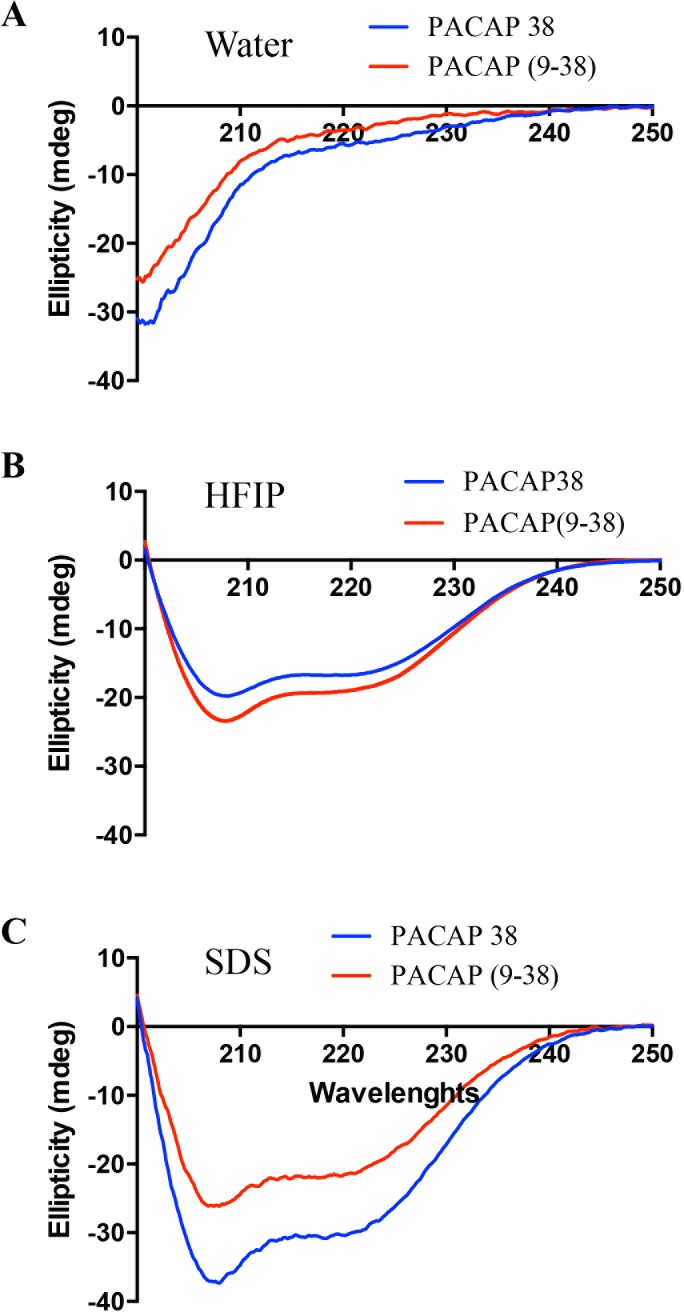
Circular dichroism analysis of PACAP38 and PACAP(9–38). Each spectrum is the mean of 3 scans corrected for solvent contribution.

The secondary structure of PACAP38 and PACAP(9–38) was investigated by circular dichroism to ensure the conservation of the C-terminal α-helix[9, 23]. As shown in Fig 2, PACAP38 and its truncated analog exhibit two negative minima at 208 and 222 nm in HFIP and 10% SDS (Fig 2B and 2C), characteristic of the presence of an α-helical structure. Conversely, in water, no structure was noticeable (Fig 2A).

**Fig 3:**
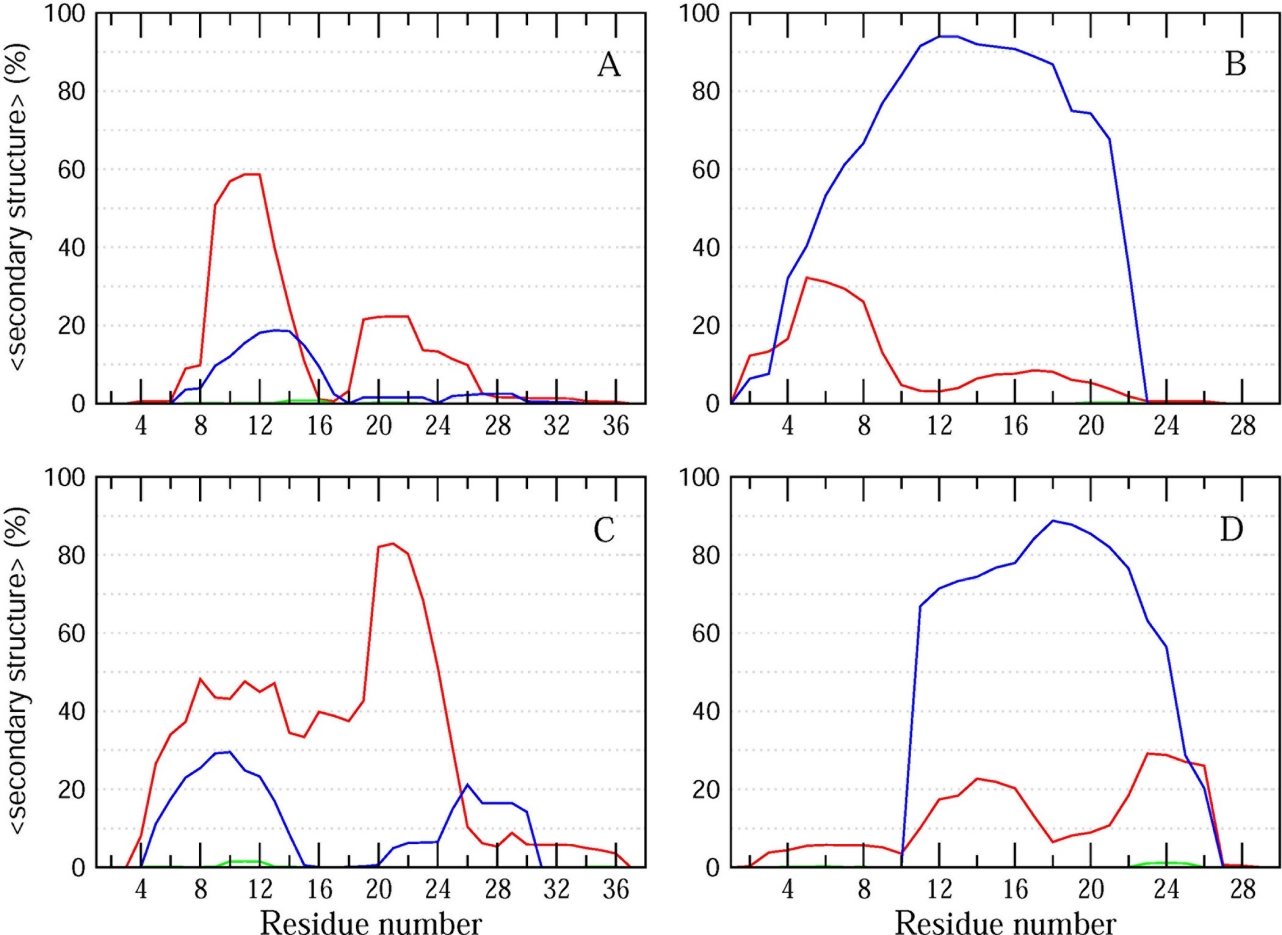
Secondary structural propensities of PACAP38 and PACAP(9–38). The average percent α-helix, 3_10_ and π helix as a function of residue number along the sequence are represented as red, green and blue lines, respectively, for two independent MD runs of PACAP38 (A, C) and PACAP9-38 (B, D).

Molecular dynamics (MD) simulations provide atomistic insights into structural properties of proteins. Here, we performed MD simulations of PACAP38 and PACAP(9–38) bound to the surface of a 1,2-dipalmitoylphosphatidylcholine (DPPC) lipid bilayer to characterize the secondary structural propensities for the two membrane-bound peptides. DPPC was chosen to mimic the zwitterionic phosphatidylethanolamine-rich membranes of gram-negative bacteria[30]. Two sets of 300 ns simulations were performed for each of the two peptides, corresponding to a cumulative simulation time of 1.2 μs. As previously demonstrated, this time scale provides a good approximation of peptide reorganization and structural behavior in solution[31]. A comparison of the secondary structural propensities of the two peptides confirms the predominant helical propensity in the two peptides (Fig 3). Interestingly, while PACAP38 showed a higher propensity for the formation of α-helical conformations relative to π- and 3_10_ helices (Fig 3A and 3C), PACAP(9–38) consistently displayed a significantly higher propensity for π-helix conformations in the two independent simulations of the peptide (Fig 3B and 3D). This enhanced propensity of PACAP(9–38) to adopt π-helix conformations may contribute towards its higher selectivity against *Burkholderia cenocepacia* J2315. We note that while MD simulations provide important insights into the differences in the conformational properties of the two peptides, further experiments such as COSY and NOESY 2D NMR experiments would be necessary to confirm these observations.

## Conclusions

We have demonstrated that the human neuropeptide PACAP38 possesses an antibacterial activity against a broad spectrum of bacteria. Notably, PACAP38 was able to inhibit the growth of the epidemic pathogen *B*. *cenocepacia* J2315 with a MIC of 10 μg/mL. Investigation about its mechanism of action suggested that a PACAP metabolite, identified as PACAP(9–38), might indeed be responsible for the observed PACAP38 antibacterial action on *B*. *cenocepacia* J2315 and *B*. *subtilis* PY79. Surprisingly, PACAP(9–38) exhibited an increased specificity toward *B*. *cenocepacia* J2315 compared to other tested bacteria, a phenomenon that might be related to its ability to cross the bacterial membrane. Indeed, differences in the bacterial wall composition[32] might be responsible for the increased selectivity of PACAP(9–38), which was generally generated *in situ* within the bacterial cytoplasm following PACAP38 entry. Accordingly, it was observed that the uptake efficacy of PACAP within eukaryotic cell cytoplasm was dependent on the expression of cell surface glycosaminoglycans[33]. Another possibility relies on the predisposition of PACAP(9–38) to adopt π-helix conformations rather than α-helical conformations like PACAP38. While the mechanisms of action remain to be completely understood, we believe that PACAP38 and related derivatives can pave the way toward the development of new therapeutic agents against multidrug resistant bacteria, and more specifically the *Burkholderia cepacia* complex.

## Abbreviations

CD: Circular dichroism
DPPC: 1,2-dipalmitoylphosphatidylcholine
PAC1: pituitary adenylate cyclase-activating polypeptide type 1 receptor
PACAP27: 27-amino acid isoform of PACAP
PACAP38: 38-amino acid isoform of PACAP
VIP: vasoactive intestinal peptide
VPAC1: VIP/PACAP type 1 receptor
VPAC2: VIP/PACAP type 2 receptor
